# Evolution of a chronic pain management program in a Northwestern Ontario community: from structural elements to practical application

**DOI:** 10.1186/s12913-022-08766-w

**Published:** 2022-11-15

**Authors:** Hadi Shojaei, Shehnaz Fatima Lakha, Ashley Lyon, Mark Halabecki, Mary Donaghy, Angela Mailis

**Affiliations:** 1 St. Joseph’s Care Group, 710 Victoria Avenue East, Thunder Bay, ON P7C 5P7 Canada; 2 Northern Ontario School of Medicine University, Thunder Bay, ON Canada; 3 Pain and Wellness Centre, 2301 Major Mackenzie Dr. West, Unit #101, Vaughan, ON Canada; 4 Division of Physical Medicine & Rehabilitation, Department of Medicine, University of Toronto, Toronto, Ontario Canada

**Keywords:** Chronic pain, Interdisciplinary program, Inter-professional, Patient-centered

## Abstract

**Background:**

Chronic pain is a highly prevalent health problem especially in rural regions. There is a dearth of comprehensive pain management programs particularly in rural areas.

**Aim:**

The objectives of this paper are to describe the evolution of an interprofessional chronic pain team employing a patient-centered model of care with a biopsychosocial approach, and health services metrics.

**Method:**

This descriptive case study approach includes an overview of the Chronic Pain Management Program (CPMP) services at St. Joseph Care Group in Thunder Bay, NW Ontario; the process involved in the development of an interprofessional chronic pain team employing a patient-centered model of care with a biopsychosocial approach; and metrics of the program’s operations.

**Results:**

Established in 1998, CPMP has evolved to become inter-professional, providing consultations and management, with partial funding by the Ontario Ministry of Health and Long term Care that has allowed expansion of services. The CPMP currently provides three distinct program streams as follows: a) Intensive 6-week, four half-days/week, outpatient program that offers an interdisciplinary team approach in groups and individual format; b) PACE-IT (Pain Assessment Collaborative Education Inter-professional Therapy), 8-week long, half-day/ week, interprofessional treatment program, in person; and c) Individual format for one-on-one services for patients not fitting in either the 6-Week or PACE-IT programs. In addition, Additional services provide virtual consultations and didactic videoteleconference sessions on opioid stewardship and pain management to health providers. Health services outcomes, research, and educational opportunities across the Northwestern Ontario Region, challenges and future needs are discussed.

**Conclusion:**

The CPMP’s model of care can serve as a foundation for expert chronic pain care delivery across rural Canada, and as template for similar institutionally-based and publicly funded pain clinics.

## Highlighted summary points

- Taken together, the current CPMP and future goals are designed to address the gaps in pain management for patients in rural settings.
- Our goal is to transform the management of chronic pain by providing seamless care at the referral, continuing care throughout the intensive programs, and care of patients once they are discharged in the community.
- The important next steps are to determine the efficacy of the CPMP in preventing chronic pain and disability and to evaluate the extent to which the CPMP reduces overall costs to the healthcare system

## Introduction

Chronic pain impacts 20% of people globally [1]. It is generally accompanied by distress, demoralisation, and functional impairment, making it a substantial cause of suffering and financial burden [2]. Chronic pain is influenced by a range of biopsychosocial factors. This understanding has led to the biopsychosocial model of pain, which is widely acknowledged as the best approach for understanding pain and guiding its treatment [3]. Currently, the standard chronic pain treatment options provided by Canadian physicians are limited to those funded by Canadian public health insurance plans (eg. medical visits, injections and medications). This is problematic because various pain management guidelines urge the use of psychological, behavioural, or less invasive physical therapies, either alone or in combination with traditional pharmaceutical treatment [4–6].

Pain medicine experts agree that the successful management of chronic non cancer pain (CNCP) requires a multidisciplinary approach [7]. Systematic reviews have shown that well-structured and organized multidisciplinary programs (MDPs) have been shown to be effective for patients with chronic pain [8]. Gatchel et al. (2014) [9], however, make a clear distinction between *multidisciplinary* versus *interdisciplinary* pain management. While both programs use multimodal treatments and various clinicians, interdisciplinary pain management programs share common rehabilitation philosophy, have robust and ongoing communication between team professionals, and active patient involvement, and are considered superior to multidisciplinary pain programs [9].

Canadian chronic non-cancer pain (CNCP) patients are treated in a number of clinical settings by various and diverse health professionals including primary care, secondary care, allied health care and dedicated pain specialists. Medical care costs (medical visits and hospital care), as well as, costs of interventional procedures (various types of injections) are covered by the provincial public health care systems, though differences may exist between provinces. In contrast, allied health care costs (e.g. psychological services, chiropractic, massage etc.), are paid out of pocket, by Extended Health Benefits or private insurances, therefore inaccessible to most chronic pain patients. The existence of numerous and variable treatment options reflect different practitioner training and philosophy, heterogeneity of patients and diagnoses, financial considerations, distance from a given clinic, availability of specific treatments, and geographic differences.

Access to chronic pain services is especially limited for vulnerable populations, such as those in rural and remote regions, indigenous populations, low income populations, and for those who have difficulties with social determinants of health [10].

Northern and rural communities have their own set of health issues, economic concerns, demographic traits, resource shortages, and cultural practises, all of which combined affect the peoples’ health [11]. In a study conducted by Jackson and colleagues [12], poverty, limited education, and limited access to good health services, are three of the most pressing concerns encountered by rural inhabitants. The authors concluded that recognition and administration of pain is crucial in resource-limited geographic places like rural areas.

Chronic pain is assumed to be more prevalent in rural areas [13]. Currently, Thunder Bay in Northwestern (NW) Ontario has the only interprofessional Chronic Pain Management Program (CPMP) at St. Joseph’s Care Group. Northwestern Ontario is 40% of Ontario’s landmass or an area the size of France, across which, a population of 300,000 is spread in small, isolated pockets. Approximately 60,000 people corresponding to 20% of the Northwestern Ontario population^1^ have chronic pain and little access to care. Developing integrated, patient-centered model of pain care in rural communities, however, is met with many challenges.

The present paper specifically outlines the background of CPMP services at SJCG in NW Ontario, the implementation and development of an inter-professional chronic pain team approach in a community-based hospital in Thunder Bay, and the practical application of a patient-centered model of care with a biopsychosocial approach, as well as health service metrics. The CPMP is designed specifically to diagnose chronic painful conditions and offered treatments in a patient-centered whole-person approach (addressing medical, psychological and psychosocial needs) in a timely manner. Since this study just contains a description of the program and services, ethics approval is not necessary. However, we did receive approval from the executive team, who all reviewed the paper.

## Overview of St. Joseph’s Care Group’s Chronic Pain Management Program in Thunder Bay

### Background

The Chronic Pain Management Program (CPMP) was established on March 24, 1998, at St. Joseph’s Hospital in Thunder Bay. At inception, the chronic pain team included Psychometrist, Psychologist, Physiotherapist, Rehabilitation Assistant, Social Worker, and Recreation Therapist. This team developed a six-week program, five days per week, eight hours per day, eventually reduced to four days per week, five hours per day, to align with available resources, utilizing also four beds on a medical inpatient floor. In 2007, the team was tasked with developing a return-to-work program and subsequently moved to an off-site location. The chronic pain team continued to provide services funded through the Ontario Ministry of Health and Long Term Care (MOHLTC), but also generated revenue through referrals from the Workplace Safety and Insurance Board and other insurance companies. Ultimately low third party referrals led to discontinuation of the insurance-based service and relocation of the team back to St. Joseph’s Hospital site. In 2011, a family physician as a pain consultant began working with the team.

In 2016 the chronic pain team partnered with two local primary care offices to provide in-house chronic pain consultation as an opportunity to build capacity within primary care and to introduce patients to the self-management model of care for chronic pain management. This outreach service increased awareness of the program but was short lived, and the team concentrated at the St. Joseph’s Health Centre site, a move that led to overall decrease in wait-time for service and increased participation in the program.

Funding for the CPMP has been provided by MOHLTC since the beginning of the program. In 2016, funding enhancements allowed for expansion of the team to include an additional Physiotherapist, Occupational Therapist, Mental Health Clinician, Social Worker, and Kinesiologist, while in 2018 a Registered Nurse position was funded.

Additionally, a physiatrist (XX, senior author) joined the team in July 2018; the latter enhanced services offered, by adding a *physician consulting service.*

### Current structure of the CPMP

The CPMP operates via a patient-focused, interprofessional, time-limited, goal-oriented team approach to address chronic pain via a biopsychosocial model of care. It aims to empower patients with chronic pain to improve their overall capacity and functioning in all parts of their lives by building coping skills and instill pain self-management principles through a patient-centred approach. Patients identify through goal setting what they would like to be able to do post-intervention and work towards achieving this with the support of the inter-professional team, they. Each patient seen in the CPMP completes a variety of questionnaires in order to determine both readiness for services and their goals for treatment.

The CPMP uses a Decision Guide (DG) (an evidence based shared-decision-making model centred on the patient). The DG (who is a clinician) along with the patient, assesses readiness, goals, and most appropriate type of treatment/program.

### Referral system

Referrals to the program have been received through the Access Point Northwest system (an online-centralized intake database system) (https://northwestaccesspoint.ca/) since May 2017, in partnership between St. Joseph’s Care Group, Thunder Bay Regional Health Sciences Centre, Alpha Court Community Mental Health & Addiction Services, and the Canadian Mental Health Association—Thunder Bay Branch. Referrals are accepted into the Central Intake Database (CID), an online database that stores patient referrals and manages waitlists. The database allows administrators and interprofessional team members to ‘move’ the patient through the stages of the program. When a referral is received, it proceeds through the following stages:

- New referral (screened for fit and appropriateness)
- Welcome Workshop
- Questionnaire Session
- Decision Guide (DG) appointment
- Internal referrals are made to appropriate discipline/service(s)

The CPMP receives referrals across NW Ontario. Patients are considered candidates if they fulfill the following criteria: are over 16 years of age, live in Northwestern Ontario, have chronic pain that interferes with daily activities, and are ready to learn and practice self-management strategies.

The CPMP offers three streams of care: (1) 6-week Program, 2) PACE-IT (Pain Assessment Collaborative Education Interprofessional Therapy), and 3) Individual Care (see program details in the description of the program) for patients to enroll.

Once the service is ready to accept a patient, an appointment slot is created and the patient is contacted. If the patient declines, their file is closed in CID. If the proposed service is accepted by the patient, the patient is moved through and remains on the service list until programming is complete. Once the patient has completed the service, the referral request is closed. In June 2019, the ability to see waitlist numbers and wait times was launched in CID-specific dashboards.

### Programs/ services offered

#### Intensive program

This is an intensive 6-week, four half-days per week (Monday to Thursday) outpatient program that offers an interdisciplinary team approach in groups and individual format, both in person and virtually during the Covid-19 pandemic to allow continuation of service due to pandemic restrictions. Over the 6-week program, patients participate in psychoeducational group lectures that are delivered by all members of the team, take part in individual and group exercise training sessions, meet with individual team members (Occupational Therapy, Psychology, Recreation Therapy, Social Work, and Physiotherapy) as agreed during the Decision Guide meeting to address treatment goals, and have a consultation with the pain physician. Each patient completing the program receives over 80 h of pain services learning self-management techniques through psychoeducational group lectures and individualized services. Each client’s schedule will be slightly different because they may have additional sessions with a particular discipline and decide that they don’t need to see another discipline. Also, they have breaks in their schedules, time that they can use for going for a walk, or additional sessions with someone, relaxing, reading etc. On average, each client attends three group sessions per day (e.g., education, movement, and relaxation) and between two and three individual sessions with therapists.

#### PACE-IT

This is an 8-week long, half-day-per week, interdisciplinary treatment program, both in person and virtually during the Covid-19 pandemic to allow continuation of service due to pandemic restrictions. This program used to be 12 weeks, but it was reduced to 8 weeks, offering one more program each year in order to decrease wait times. During the PACE-IT program, patients are learning self-management techniques, mainly through psychoeducational group lectures delivered by all members of the team, as well as group exercise sessions. They are also meeting with individual team members (Occupational Therapy, Psychology, Recreation Therapy, Social Work, and Physiotherapy) as decided during the Decision Guide meeting to address treatment goals, and have a consultation with the pain physician. This program was introduced in 2014 as a less intense form of treatment to accommodate patients who are not ready or cannot commit to a full 6-week program, or who have other barriers such as employment or childcare issues, among others.

Each PACE-IT patient who completes the program receives a minimum of 20–24 h of pain services throughout their journey. Each day, the patients have at least two one-hour group education sessions and 30 min of exercise/movement. For those patients who have set goals with individual team members, the sessions are scheduled outside of the PACE-IT Tuesday afternoon. Depending on their goals, patients could meet with each discipline, one or multiple, as there is no limit, but typically, they attend individual sessions while they are currently attending PACE IT, and on average, they have 2–3 sessions with each discipline. If they need more time to work on their goals, after PACE-IT is over, they will move into the “individual care stream”.

#### Individual care

If a patient does not fit in either the 6-Week or PACE-IT programs, or does not feel they would benefit from either program at the time of their decision guide appointment, he/she can be referred to the individual care stream for one-on-one services. For instance, a patient can be referred to one or more members of the interprofessional team (Physiotherapist, Psychologist, Recreation Therapist, Social Worker, Occupational Therapist, Exercise Specialist, or Pain Specialist Physician), without attending the psychoeducational group lectures or exercise sessions. They would still be offered coping strategies and learn self-management strategies catered to their individual needs.

Communication between team members for all 3 programs, is frequent and includes formal team rounds, where all providers and physicians discuss patients from the 6-Week and PACE-IT programs, as well as select patients with complex pain issues from the individual care stream.

In 2018, a new pain physician (specialized in Physical Medicine and Rehabilitation who had completed a chronic pain management program fellowship), joined the CPMP team as a consultant (HS, senior author). This consultant created a physician consultation service for patients requiring medication review, diagnostic pain assessments, and further therapeutic recommendations. Because of this service, the program now receives an increasing number of referrals from family physicians or nurse practitioners requesting pain medication management including opiates, and clarification of the diagnosis. A registered nurse was hired in 2019 to support the work of the physician consultant with MOHLTC funding obtained in 2018.

A breakdown of the interprofessional team can be found in Table 1: Service Provisions at the CPMP outlining the average number of patients seen in each program and decision guide appointments offered in an average week.

**Table 1:** Service Provisions at the CPMP

**Service Distribution**	**Number of Patients per week/service**				
**Discipline**	**6 Week Program**	**PACE IT Program**	**Individual Care**	**Total hrs./week**	**Number of Decision Guide**
Physio# 1	8–15	1–2	1–3	10–20	1
Physio# 2	-	4	16	20	-
Social W# 1 HBSW	12	-	-	10	4
Social W# 2 MSW	0–2	2–4	6–10	8–16	4–6
Psychotherapist	5–6	3	10	18–19	1
Ph.D.-Psychologist	5–6	3	12	20–21	-
Psychological associate	-	2	18	20–21	1
OT# 1	3	7	7	17	1
OT# 2	10–14	4–5	-	14–19	2
Exercise Specialist	6–8	8–10	6–8	20–26	2
Physician	1–4	3–6	5–10	15–24	-
Dietitian	8–10	8–10	4–7	20–27	-
Recreational Therapist	3	2	8	13	-
Total	63–83 patients	49–58 patients	94–109 patients	205–253	16–18 patients
Total Number of Hours for Provision of Services in Programs	72 h	30–36 h	As needed	-	-

The average number of patients seen in each program and decision guide appointments offered in an average week

### Facility description

The CPMP is located on the first floor of St. Joseph’s Health Centre North and includes a reception area, medical and administrative offices, treatment rooms, a gym, a boardroom, and a shared office space, with approximately 4600 square feet of space. Application for more funding has been submitted to MOHLTC in order to obtain additional office space. CPMP is now operating as a paperless facility with an electronic medical records system safeguarded by a cloud-based company.

### Funding

The CPMP services are funded as follows: a) Medical consultations and medical management are remunerated by OHIP; b) patients after motor vehicle or work related accidents are funded by third parties (such as auto insurance, WSIB, etc.); while c) interdisciplinary pain services and support staff are funded by MOHLTC.

### Staff

The CPMP has always functioned as an interprofessional team and overtime the team has expanded. Currently, the CPMP staff consists of a manager; two physicians (one family medicine, and one specialist in physical medicine and rehabilitation); and allied health professionals, namely one psychologist, one psychological associate, two physiotherapists, a dietitian, two occupational therapists, two exercise therapists, one registered nurse, one rehabilitation assistant, one recreation therapist, two social workers, one psychotherapist, a half time data analyst, and a program administrator.

All of our physiotherapists and exercise therapists are strength trainers and have additional expertise in postural analysis, body mechanics, and rehab exercise training. The social workers place emphasis on social determinants of health and provide counselling and support for individuals. The dietitian places emphasis on nutrition, weight management, and life choices. The psychologist, psychological associate, psychotherapist, and MSW provide psychological consultation and support and a variety of psychological therapies, including mindfulness, and cognitive behavioural therapy.

The CPMP staff members use a variety of therapeutic equipment in the gym located at St. Joseph’s Health Centre North to assess patient’s baseline function and measure improvement of physical functioning, while the Occupational Therapists’ equipment and supply needs relate mostly to assessing and prescribing assistive devices (i.e. demo walkers, braces, splints) as well as the manufacturing of splints and custom orthotics.

All members of staff are offered access to CPMP-funded continuous health education, including presentations and attendance at our annual Canadian Pain Conference meetings. Additionally, in order to maintain a highly engaged and satisfied workforce, St. Joseph’s Care Group conducts employee and physician surveys every two years asking the staff to provide feedback and suggestions regarding their experience in their workplace.

### Patient flow

Referrals for medical consultation and requests for assessment and treatment are made through the Access Point Northwest website and reviewed in CID by medical and administrative staff to ascertain that basic information has been received.

During the 2020/21 Covid-19 pandemic, patients were provided with an online Welcome Workshop and phone intake prior to placement on wait lists for admission to the program. Prior to the pandemic restrictions, a three step resource and time intensive process was utilized; however, the amount of in-person contact has been reduced substantially now with the online process. All referrals continue to be received and reviewed prior to the clerical staff contacting the patient and providing a link to the online Welcome Workshop. The online Welcome Workshop is a PowerPoint presentation with a voice recording which can be accessed in the privacy of the patient’s home; this presentation is identical to the previous in-person session. Subsequently, the Decision Guide explains the program expectations during their appointment, (in-person or over the telephone), where discussion about treatment goals, readiness for treatment, and a personalized treatment plan is developed. The Decision Guide then makes the referral to the most appropriate program (6-Week, PACE-IT, or Individual Care) within the CID.

**Fig. 1:**
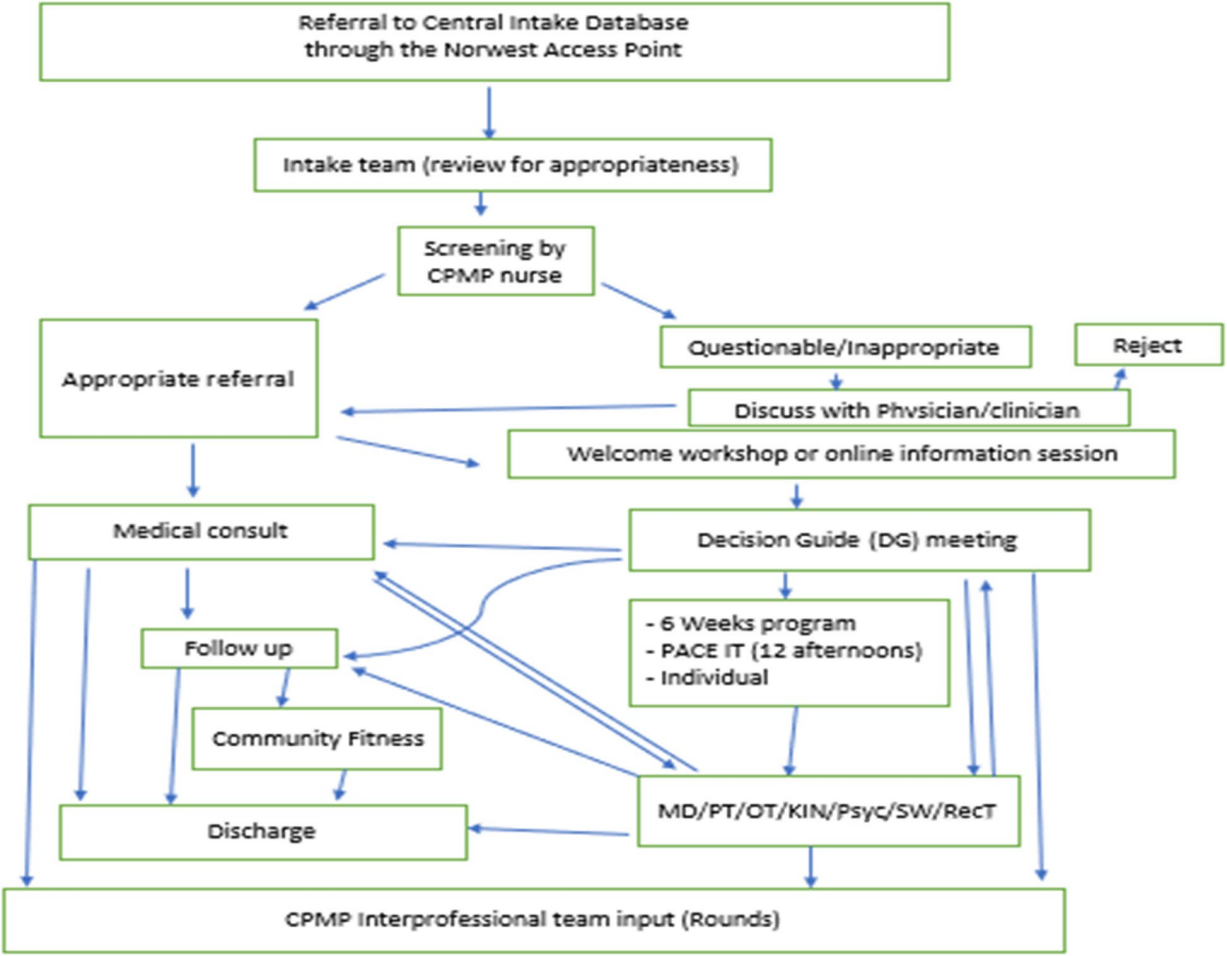
CPMP Patients Flow Chart

Of note, this new process has substantially reduced the number of no-shows for Decision Guide appointments and the wait time for access to services, while it emphasizes timely discharge after completion of services, as more focused treatment goals are being determined at the Decision Guide appointments (Fig. 1).

### Partnerships with community

Prior to 2020, Community Outreach was an extension of the chronic pain program in two communities, the Manitouwadge Family Health Team and Atikokan Hospital. These partnerships have since concluded, and their patients continue to be referred to the CPMP for services.

Chronic pain services until 2018, offered a condensed program specifically for regional patients. Patients residing outside of Thunder Bay attended a condensed and intensive program model over 4 days offered at St. Joseph’s Health Centre. Prior to the pandemic, planning for this regional sessions was extremely resource-intensive, consuming many hours of clinician time coordinating schedules and appointments, and often resulted in a low attendance. Currently, the program continues to accept referrals from all of NW Ontario and provide services as best as possible in a hybrid model of care (virtual and in-person). Some of the challenges still facing patients referred from regional communities include: complex medical history and significant functional impairment impacting ability to travel, financial issues limiting access to travel and pay for lodging, lack of community supports, and continuity of care.

### Virtual services and ECHO services

In 2013, St. Joseph’s Care Group became involved in the launch of the Extension for Community Healthcare Outcomes pilot project, known as “Project ECHO: St. Joseph’s Care Group—Chronic Pain & Opioid Stewardship”. The ECHO model consists of a Hub and Community Partners. The Thunder Bay Hub (https://sjcgecho.squarespace.com/) is located at St. Joseph’s Health Centre and is comprised of CPMP allied health clinicians and one MD who serve as consultants in the didactic presentations and share knowledge through case-based learning. This is followed by a discussion with registered participants of ECHO (learners) who connect virtually from communities across Canada, sharing knowledge and insights and are referred as the Community Partners.

Ontario Telehealth Network, Cisco WebEx Meetings, and Zoom have been utilized to integrate regional patients into CPMP at SJCG, as well as to accommodate patients due to pandemic restrictions. These partnerships and educational opportunities are vital to providing timely and effective service and support to patients in the region, reducing wait times and eliminating the need for travel, where possible. Of note, with the growing need for chronic pain services, other communities have reached out to our chronic pain team for support and guidance in developing services within their communities.

### Health services outcome measures

Several validated multi-dimensional measures of physical, psychological and social function, as well as various pain and other symptoms, are collected from our patients. Details, however, of validated batteries and patient-reported outcomes (see below) will be the subject of subsequent papers, as the present paper focusses on the description and evolution of pain services at SJGG in NW Ontario.

In regard to batteries of tests, before COVID, patients used to complete a number of specialized questionnaires as follows: After the Welcome Workshop and before the Decision Guide appointment, patients complete several validated measures such as: Brief Pain Inventory (BPI), Pain Catastrophizing Scale (PCS), Tampa Scale of Kinesiophobia, Pain Stages of Change Questionnaire, and Multidimensional Health Locus of Control. The patients fill out the same upon discharge. However, as the result of the Covid-19 pandemic, the number of outcome measures has been reduced temporarily, and St. Joseph’s Care Group has started to use RedCap to capture the data. Patients are now provided with a link at the time of booking their Decision Guide appointment and asked to complete only the Brief Pain Inventory (BPI) and the Tampa Scale of Kinesiophobia (TSK) online through RedCap. This information is then saved to the RedCap database, to facilitate outcome measure reporting to the MOHLTC and to guide CPMP’s future planning purposes.

The Table 2 below provides a collective view of the changes in wait times and number of referrals over time between January 2018 and March 2020, prior to Covid-19 pandemic measures.

**Table 2:** Program Metrics

Metric	January – March 31, 2018	January – March 31, 2019	January – March 31, 2020
Number of new patients seen in the program	159 referred, 65 attended	287 referred, 114 attended	195 referred, 144 attended
Number of outpatient clinics held	4 Welcome Workshops (info sessions)	7 Welcome Workshops (info sessions)	Welcome Workshop (information session) transferred to on-line format for individual viewing
Average wait time from referral date to Welcome Workshop (info session) availability	61.79 days	70.96 days	13.83 Days
Average time from Welcome Workshop to Decision Guide appointment	22.68 days	22.35 days	27.66 days
Average time from Decision Guide appointment to intervention initiation	88.68 days	75.49 days	54.47 days
Average time from referral date to intervention initiation	173.15 days	168.80 days	101.50 days
Number of patients receiving treatment in the program^a^	275	330	472

^a^The number of new patients receiving treatment at any given time consists of new patients plus existing patients in a given program

### Teaching and research output

The CPMP staff welcomes several learners through such institutions as the Northern Ontario School of Medicine medical students, Royal College Anesthesiology residents, Family Medicine residents, Ph.D. Psychology residents, Kinesiology students, Occupational Therapy students, and Psychotherapy students. In addition, we have had site visits from nurse practitioners and physiotherapists from a few regional communities to gain further knowledge in chronic pain education delivery and processes.

In regards to research, CPMP has access to the research ethics board of St. Joseph’s Care Group and can make grant submissions to the Northern Ontario Academic Medicine Association (NOAMA). For current research projects, the data is extracted partially through the electronic medical records system and through separate databases maintained by various team members.

Several abstracts (and posters) were presented at various conferences and meetings, such as the Canadian Pain Society in 2018, 2019 and 2020 [14–16]. CPMP is in the process of submitting papers for peer-reviewed publications. Ethics approval for this project has been obtained from the St Joseph’s Care Group Research Ethics Board on December 4, 2020 (Protocol # 2,020,005). We are preparing further papers for future submission relating to the characteristics of our chronic pain population and effects of our pain management treatments.

### Challenges

The following represent challenges to CPMP’s current function:

- Long wait times: Currently wait time is 101.50 days from referral date to intervention initiation due to program processes and increases in the numbers of new patient accepted into the programs. A wait list challenge (once accepted in the program) is appointments with a clinician. For example, the waiting list for an appointment with the dietitian, is about 10 months for individuals wanting nutrition counseling.
- Coordinating patient care and programming. The no-show rate for patients residing in Thunder Bay, as well as outside Thunder Bay, is high. For example, the March 2019 regional session, had a No Show rate of 62.5%, due to significant functional impairment affecting ability to travel, financial issues, and lack of community supports in sustaining treatment gains once returning home.
- Opioid medication management at CPMP is a challenge given serious opioid crisis in Thunder Bay and many communities in the region. Currently, the CPMP is not set up or aligned with opioid management or tapering, although the physiatrist is doing his best to take care of patients with no family physician or when the family physician is unable or unwilling to prescribe or address opioids (a problem unfortunately of universal dimensions across Canada).. Unfortunately, while inclusion of other allied health care professionals such as pharmacists, may help, it will not address the above cited problems.

### Future medically focused goals

The CPMP will continue to work on its present services, but plans to expand. The CPMP Physician consultant will initiate regional visits yearly, especially for those who have already started pain management services, in order to increase capacity in the region, in closer collaboration with regional partners, and possible satellite services. Additionally, the CPMP aims to build further links with community primary care providers and specialist groups to disseminate evidence-based knowledge on chronic pain diagnosis and management.

In regard to future research endeavours, the CPMP expects to start two new research projects:

- A cross-sectional descriptive study regarding community populations that attend the CPMP in Thunder Bay;
- An Academic Health Sciences Centre Alternative Funding Plan project of MyPainMyRecord, to explore how to include the patient voice in person-centred care for greater autonomy, engagement, agency, and empowerment regarding their pain care;
- The physician consultant and allied health clinicians at CPMP are currently designing shared primary care models and to provide OTN services for regional support, as well as (in collaboration with academic managers at NOSM) teach medical students and residents multiple modules such as Structured Clinical Skills (SCS), Whole Group Sessions (WGC), and other relevant topics.

## Discussion

This paper presented in detail the structure and evolution of a chronic pain management program in Northwestern Ontario. The CPMP offers multimodal and interprofessional care to chronic pain patients, coupled with educational and research output and fulfills nearly all of the following principles of the Ontario Ministry of Health comprehensive pain strategy as follows: It is interprofessional and multimodal; patient centred; operates within the chronic disease management framework; provides evidence-based and stepped-up care; accountable to MOHLTC in regard to the services paid by taxpayer’s funds and the outcomes of these services; provides education for both patients and health providers; shows continuous quality improvement; supports data information exchange and research; and offers reasonable access to the care continuum.

In order to overcome existing challenges and improve further its services, the CPMP is in need of additional resources as follows:

- The addition of a pharmacist to the program could facilitate some of the medication related issues with chronic pain patients and provide assistance to the pain physician in that regard.
- Attracting well trained pain physicians to the program (though appropriate incentives in order to join rural/ Northwestern Ontario practices) is key to the maintenance and expansion of all services as well as educational and research output.
- Hiring additional allied health practitioners would provide opportunities to extend services within Thunder Bay and regionally.
- Introduction of electronic consultations through OTN and support of shared care models will improve wait times. Additionally, the CPMP team can mentor professionals in small towns through virtual connections, in person visits, and ongoing support through ECHO, so that they can offer services to patients who cannot travel to Thunder Bay. OTN service expansion requires support for an OTN dedicated room with an exam bed, small table and chairs, OTN set-up and TV/camera, and dedicated technician for scheduling and technical support
- Recruitment of research staff is very important to accelerate the creation of a Northern Ontario Chronic Pain Research group.

## Conclusions

CPMP demonstrates the advantages of interprofessional pain management program in Northern Ontario and clearly demonstrates the feasibility of the model. However, none of the above could have materialized if it were not for sustainable funding and support provided by the Ministry of Health and St. Joseph’s Care Group.

Detailed medical consultations can assist with diagnosis, provide proper pharmacology interventions, and offer advice to referring primary care providers.

When it comes to treatment, our selection criteria place emphasis on patient goals and assessment of readiness during the Decision Guide appointment, leading to selection of those who are more likely to improve and capitalize on the interdisciplinary resources of pain management. Such selection is important since public funding is not unlimited.

CPMP presents a model and a template for similar institutionally-based and publicly funded pain clinics in other parts of Northern Ontario to combat the burden of chronic pain. In particular, CPMP’s ability to provide effective pain care in Northern Ontario has wide implications for the MOHLTC policymakers who endeavour to fund community pain management clinics, in their efforts to reduce personal suffering and the economic burden of chronic pain. Our next step will be to determine the effectiveness of our program in treating chronic pain and reducing disability and health care costs.

## Data Availability

The datasets generated during and/or analyzed during the current study are not publicly available due to institutional privacy and confidentiality, but are available from the corresponding author on reasonable request.
